# Understanding biopsychosocial factors influencing cigarette smoking among transgender and gender diverse individuals living in Argentina: a qualitative study

**DOI:** 10.1186/s12939-025-02563-7

**Published:** 2025-07-07

**Authors:** Romina Caballero, Estefania Panizoni, Raúl Mejía, Ana Zeltman, Nadir Fernanda Cardozo, Alixida Ramos-Pibernus, Ash B. Alpert, Ana Paula Cupertino, Francisco Cartujano-Barrera, Ines Aristegui

**Affiliations:** 1 Research Department, Fundación Huésped, Buenos Aires, Argentina; 2 Department of Ambulatory Care, Universidad de Buenos Aires, Buenos Aires, Argentina; 3 Asociación de Travestis, Transexuales y Transgéneros de Argentina, Buenos Aires, Argentina; 4 School of Behavioral and Brain Sciences, Ponce Health Sciences University, Ponce, PR USA; 5 Division of Hematology, Department of Medicine, Yale School of Medicine, New Haven, CT USA; 6 Department of Surgery, University of Rochester Medical Center, Rochester, NY USA; 7 Department of Public Health Sciences, University of Rochester Medical Center, Rochester, NY USA

**Keywords:** Transgender individuals, LGBTQ, Biobehavioral model, Smoking, Argentina, Community-Based Participatory Research

## Abstract

**Background:**

The prevalence of cigarette smoking among transgender women in Argentina is 42%, more than double the rate compared to cisgender women.

**Objectives:**

To understand biopsychosocial factors influencing cigarette smoking among transgender and gender-diverse (TGD) individuals living in Argentina.

**Methods:**

Between December 2023 and January 2024, 19 qualitative semi-structured interviews were conducted face-to-face with TGD individuals. TGD peer navigators recruited participants using community-based strategies. The biobehavioral model of nicotine addiction was the theoretical framework for the qualitative analysis.

**Results:**

we identified important biological, psychological, and social factors. Biological factors included: (1) Negative impact of cigarette smoking on health (e.g., medical gender affirmation), and (2) nicotine dependence. Psychological factors included: (1) Emotions (e.g., loneliness), (2) psychological disorders, (3) coping strategies (e.g., gender minority stress), (4) cognition and beliefs (e.g., psychological gender affirmation), (5) routine behavior, and (6) substance use. Social factors included: (1) social influence, (2) social acceptance of smoking, (3) social support, (4) gender-based stigma, discrimination, and violence, and (5) contextual factors (i.e., work environments).

**Conclusion:**

These results provide important insights to be considered when developing a tailored smoking cessation intervention for TGD individuals living in Argentina.

## Background

Argentina has one of the highest cigarette smoking rates in Latin America (22%) [1]. In Latin America broadly and Argentina specifically, cigarette smoking is highest among vulnerable groups [2], including those with limited financial means (i.e., low socioeconomic status) [3] and limited access to education [4]. Transgender and gender diverse (TGD) individuals often experience structural barriers to employment and education. These structural barriers contribute to the 42% prevalence of cigarette smoking among transgender women (TGW) living in Argentina [5], double the rate observed among cisgender women [2]. For example, among TGW in Argentina, education level of less than high school is positively and independently associated with current cigarette smoking [5].

Worldwide, cigarette smoking among TGD individuals has been associated with education, employment, housing, and financial means along with engagement in sex work [5, 6]. Furthermore, stigma and discrimination experienced by TGD individuals as well as other substance use have been associated with cigarette smoking [5–10]. Additionally, legal and social gender affirmation has been associated with cigarette smoking [9, 11, 12].

Despite the growing body of research on smoking cessation among lesbian, gay, bisexual, transgender, and queer (LGBTQ+) individuals around the world [13], evidence-based interventions specifically tailored to TGD individuals do not exist. This study aimed to understand biopsychosocial factors influencing cigarette smoking among TGD individuals living in Argentina. Findings will guide the development of a smoking cessation intervention tailored to TGD individuals living in Argentina.

## Methods

### Study design

We conducted a qualitative study with semi-structured interviews to understand biopsychosocial factors influencing cigarette smoking among TGD individuals living in Argentina. A community advisory board of nine TGD individuals (four transgender women, three transgender men, and one non-binary [NB] individual) guided the study design, implementation, and evaluation.

### Participants and sampling

Participants self-identified as TGD, were 18 years of age or older, currently smoked cigarettes, and were willing to participate in a 60-minute face-to-face interview. Participants were compensated with $10 USD for their time.

Purposive sampling was employed. We set an initial sample size of 17 participants, following the guidelines proposed to reach thematic saturation [14].

### Recruitment

Participants were recruited by TGD peer navigators using community-based strategies [e.g., study presentations at community-based organizations, social media (i.e., Facebook^®^ and WhatsApp^®^). TGD peer navigators conducted eligibility and scheduled a face-to-face interview among those who were deemed eligible. Recruitment continued until at least 17 interviews had been completed or until the predefined two-month period established in the research protocol was reached.

### Theoretical model

The interview guide was informed by the biobehavioral model of nicotine addiction, which states that cigarette smoking is influenced by biological, psychological, and social factors [15]. Biological factors encompass physiological and genetic influences, such as neurochemical processes and comorbidities. Psychological factors encompass mental and emotional influences, such as stress and personality traits. Social factors encompass the external social environment and cultural context, such as cultural norms, contextual triggers, social influence, and social support.

### Data collection

Between December 2023 and January 2024, R.C, A.Z., and I.A. conducted the semi-structured interviews. The interviews were conducted in Spanish, audio-recorded, and transcribed. Examples of the interview questions are: *What are your thoughts on the benefits and risks of smoking?* (biological factors); How do you feel when you smoke? (psychological factors); and *In what situations are you more likely to smoke?* (social factors). Participants also completed a sociodemographic survey that included questions about gender identity, age, level of education, place of birth, occupation, and engagement in sex work. Moreover, the survey included cigarette smoking-related variables, for example the age of participants’ smoking initiation, smoking patterns, time to first cigarette [16, 17], type and number of cigarettes smoked per day, and previous quitting attempts.

### Data analysis

Qualitative data analysis was guided by the biobehavioral model of nicotine addiction using thematic analysis to inform the systematic identification, analysis, and reporting of patterns within the data [18]. Using NVivo version 14.23.1 [19], R.C. and E.P. independently coded the first three transcripts, creating inductive codes as emergent topics arose. Then, R.C. and E.P. compared codes and, under the supervision of I.A., resolved discrepancies and created a codebook. R.M., A.P.C., and F.C.B. reviewed, provided input, and approved the final codebook. Using the codebook, R.C. and E.P. coded the rest of the interviews, iteratively revising as they went and choosing illustrative quotes for each theme. E.P. translated the illustrative quotes into English, and I.A. reviewed and provided feedback on the translations, which were revised accordingly. Means, medians, and frequencies were calculated for sociodemographic and cigarette smoking-related variables using the Statistical Package for the Social Sciences v29.0 [20].

### Positionality

R.C. is a doctor in psychology and E.P. is a medical doctor; they are cisgender research fellows committed to advance tobacco control and transgender health. I.A. is a doctor in psychology and a cisgender researcher with extensive experience in community-based participatory research with TGD individuals. R.C., E.P., and I.A. were born and raised in Argentina, and currently live and work in Argentina.

## Results

### Baseline characteristics

Nine participants were transgender women (47.40%) and ten were transgender men (52.60%). There were no non-binary individuals. Mean age of participants was 36.37 years (SD = 10.22). All participants were born in Argentina; most (88.90%) have migrated to Buenos Aires from other parts of the country. Almost one third of participants (31.60%) had not completed high school, and 78.90% were currently employed, including 21.10% who were currently engaged in sex work. The average age of smoking initiation was 14.53 years (SD = 3.19). Most participants (78.90%) smoked cigarettes daily, with 94.10% primarily using manufactured cigarettes. Almost half of participants (47.10%) used cigarettes with flavor capsules; none reported using electronic cigarettes (Table 1).

**Table 1:** Baseline characteristics of participants (*n* = 19)

	Total* n = 19
**Characteristics**	
Age, Mean (SD)	36.37 (10.22)
Gender identity, n (%)	
Transgender women	9 (47.40)
Transgender men	10 (52.60)
Age of gender identity expression, Mean (SD)	12.38 (7.02)
Education level, n (%)	
Less than high school	6 (31.60)
More than high school	13 (68.40)
Internal migration, n (%)	
No	2 (12.10)
Yes	16 (88.90)
Currently working (included sex work), n (%)	
No	4 (21.10)
Yes	15 (78.90)
Currently engaged in sex work, n (%)	
No	15 (78.90)
Yes	4 (21.10)
Age of smoking initiation, Mean (SD)	14.53(3.19)
Smoking pattern, n (%)	
Non-daily	4(21.10)
Daily	15(78.90)
Cigarette type, n (%)	
Manufactured cigarettes, (yes)	18(94.10)
Hand-rolled cigarettes (yes)	2(13.30)
Cigarettes with flavored capsules (yes)	8(47.10)
Attempted to quit smoking in the previous year, n (%)	
No	4(22.20)
Yes	14(77.80)
Time to first cigarette, n (%)	
Within 5 min	4(23.50)
6–30 min	4(23.50)
31–60 min	1(5.90)
More than 60 min	8(47.10)

Note:

* Total may differ due to non-response on some questions

## Biological factors

Participants reported two broad categories of biological impacts of cigarette smoking: (1) the negative impact of smoking on health (i.e., general health, medical gender affirmation, the impacts of second hand smoking on health) and (2) nicotine dependence. Select quotes from participants are shown below.

### Negative impact of cigarette smoking on health

*General health.* Lung health was a prominent concern for participants, with many (*n* = 14) worrying about developing lung diseases (e.g., asthma or lung cancer).

*“I know it [smoking] is bad for me. I have two friends with cancer*, *so I know it can cause me some problems.*” (Participant 16, transgender man, 28 years old).

Five participants were concerned about cardiovascular health, perceiving that people who smoke “*are more prone to cholesterol issues*,* heart disease…and* [issues with] *blood circulation.”* (Participant 05, transgender woman, 47 years old).

Four participants noted the negative impact of smoking on their oral health and skin (e.g., dryness, blemishes, and premature aging):

“*My skin feels dry. I’ve read that [smoking] also damages your skin and [causes] other [*problems] *like bad breath*,* yellow teeth.*” (Participant 14, transgender man, 26 years old).

Six participants expressed concern that they might gain weight if they quit smoking.

*“Quitting smoking worries me because I’m afraid of replacing it with food and starting to gain weight.”* (Participant 10, transgender man, 47 years old).

*Medical gender affirmation.* Participants expressed concerns about the impact of smoking on their health in the context of gender affirmation or gender affirmation as an impetus for quitting. Four participants highlighted the potential health risks of the interaction of cigarette smoking with hormone therapy. Participants also emphasized the importance of quitting smoking to reduce associated risks:

*“It would be important for [TGD individuals] to quit smoking because we are*,* with hormone therapy*,* more prone to [thrombosis].”* (Participant 17, transgender woman, 38 years old).

“*Raising awareness about health and focusing on the consequences [of the interaction between smoking and hormones] is important.*” (Participant 10, transgender man, 47 years old).

In addition, five participants noted the need to quit smoking before undergoing gender-affirming surgeries and considered surgeries to be extrinsic motivators for quitting:

*“[I quit smoking for a while] when I started transitioning.… I had surgery*,* the mastectomy*, *and in mid-2020*,* 2021*,* well*,* I also started with testosterone.”* (Participant 11, transgender man, 22 years old).

*“Last year I stopped smoking*,* so my medical tests would come out well…. Quitting smoking helped me with…the masculinization process.”* (Participant 16, transgender man, 28 years old).

*Secondhand smoke.* The effects of secondhand smoke on the health of people (n = 12) and pets (n = 7) were described as social and environmental motivations for quitting:

[Smoking is bad] f*or my health*,* for the environment and for the people who [my smoking] damages…a plant*,* a dog*,* a human.”* (Participant 05, transgender woman, 47 years old).

”*Animals really suffer. [Cigarette smoke] causes allergies*,* affects their fur.*” (Participant 16, transgender men, 28 years old).

### Nicotine dependence

Many participants described their physical dependence on nicotine dependence and the ways it impacts their lives. Sixteen participants described a constant and often overwhelming need for cigarettes.

*“I don’t feel free with cigarettes. It’s like they control me. The desperation when you don’t have cigarettes… It is horrible. The level of addiction it generates in me is like… wow. I can’t believe it. It’s a lot.”* (Participant 19, transgender woman, 44 years old).

Physical symptoms of abstinence included sweating hands and tachycardia.

*“[If I do not smoke*,* I feel] nervous. I get grumpy… super anxious in every way. So it’s not that I like it*,* but I think it’s addictive. That is all.”* (Participant 14, transgender man, 26 years old).

Some participants described smoking in the middle of the night, asking strangers for cigarettes, or reusing cigarette butts.

*“There was a moment*,* when I didn’t have one [cigarette]*,* that I would pick up butts or unfinished cigarettes from the floor just to smoke them.”* (Participant 03, transgender woman, 37 years old).

## Psychological factors

Participants reported six psychological factors associated with cigarette smoking: (1) emotions (i.e., emotional states, loneliness), (2) psychological disorders (i.e. depression, chronic anxiety), (3) coping strategies (i.e., stressful life events, gender minority stress, healthier emotional regulation strategies), (4) cognition and beliefs (i.e., psychological gender affirmation, willpower), (5) routine behavior, and (6) substance use.

### Emotions

Participants identified a range of emotions as primary triggers for smoking, which participants used to manage emotions and overwhelming mental states.

*Emotions as Triggers*. For most participants (*n* = 15), cigarettes were frequently used as an emotional regulator to cope with intense emotional states like anxiety, stress, tension, and nervousness:

*“[Smoking is] also an emotional thing*,* right? When facing anxiety*,* a cigarette is a good partner.”* (Participant 10, transgender man, 47 years old).

*Loneliness*. Seven participants explained that loneliness, often due to a lack of social network (e.g., family, TGD community) or being alone (e.g., after work), was a reason they smoked, and that cigarettes became a form of companionship.

*“I feel that I am alone*,* and… it’s like it’s [smoking is] a companion for me (…) I see it [a cigarette] light up*,* I see the smoke [and]…it’s like a companion”* (Participant 05, transgender woman, 47 years old).

*“When I’m waiting for [a sex work client]…[the cigarette] is there keeping me company.”* (Participant 12, transgender man, 21 years old).

### Psychological disorders

Five participants described that smoking provided a way to manage overwhelming emotions and thoughts associated with mental health conditions like depression, chronic anxiety, and panic attacks.

One participant explained that he smoked to mitigate intrusive thoughts of self-harm:

*“Smoking is bad. It was bad. It continues to be bad*,* but it helped me with the intrusive thoughts. Depression and autism give me panic attacks; instead of hurting [myself*,* I] light a cigarette. Smoking calms the rumination.*” (Participant 16, transgender man, 28 years old).

Conversely, one participant reported smoking less during depressive episodes:

*“When I was in a depressive episode almost a year ago*,* all I did was sleep*,* sleep*,* sleep*,* and I didn’t smoke. I mean*,* depression doesn’t just make me sleep… I don’t even feel like smoking.”* (Participant 17, transgender woman, 38 years old).

### Coping strategies

Participants described managing various specific stressors through smoking, using it as a coping strategy to relieve temporary emotional instability.

*Stressful life events.* Fourteen participants described smoking as an avoidance coping strategy to regulate emotional instability and provide temporary relief from a variety of stressful life events. Commonly mentioned stressors included interpersonal conflicts, financial or work-related challenges, death of loved ones, and academic pressures.

*“…Personal issues*,* work*,* whatever…When I have problems*,* my scapegoat is smoking.”* (Participant 06, transgender man, 56 years old).

*“When I get this feeling*,* an anxiety that something major has happened in the family*,* I start crying*,* and then*,* well… I light up a cigarette.”* (Participant 07, transgender man, 39 years old).

*“I got nervous because I [hadn’t done sex] work rent was due soon. I was nervous*,* and you know [I smoke].”* (Participant 17, transgender woman, 38 years old).

*Gender Minority Stress*. Six participants reported chronic stress related to transphobia, which led some to smoke as a coping strategy to endure the emotional strain:

*“When [people] insult you*, ‘This f*cking maricón [colloquialism for gay],’ ‘this f*cking torta [colloquialism for lesbian],*’ or ‘this travesty [colloquialism for transgender women].’…. You light up a cigarette to get through the hard times… (…) Walking down the street is different for a trans person*,* more so for trans women.”* (Participant 07, transgender man, 39 years old).

*“And sometimes problems lead you to want to smoke more*,* and usually transgender people have a slightly more hostile or complex life. I’m not saying that [cisgender] people don’t have it too*,* but I feel that these things usually happen because of discrimination.”* (Participant 14, transgender man, 26 years old).

### Cognitions and beliefs

*Psychological gender affirmation.* Some transgender men (*n* = 2) reported that smoking made them look and feel more masculine:

*“Before*,* I was compelled to be her*,* to behave like a woman. Unconsciously*,* I tried everything possible to make myself appear masculine and I realized that smoking [made me look more masculine]”* (Participant 16, transgender man, 28 years old).

*Willpower.* Nine participants believed that quitting smoking was a personal decision determined by self-commitment and some of them participants suggested that smoking cessation treatments were ineffective:

*“I wasn’t putting much willpower into quitting smoking*,* and I think that If I failed [to quit]*,* then the [smoking cessation] app [I was using] failed.”* (Participant 11, transgender man, 22 years old).

*“The person who really wants to quit [smoking]*,* will quit. The person who wants [to quit smoking] is going to go through a process*,* quit*,* and that’s it.”* (Participant 18, transgender woman, 35 years old).

### Routine behavior

Smoking became deeply embedded in participants’ daily routines, tied to activities like breakfast, drinking mate, a traditional Argentine beverage, and waiting for transportation. Fourteen participants reported that these routines compelled them to smoking as an automatic behavior:

*“It’s a habit. I see it as just another part of my life.”* (Participant 09, transgender men, 32 years old).

*“I get up. I smoke a few cigarettes. I have a coffee and I smoke a cigarette. After eating*,* I smoke a cigarette. I smoke another one*,* brush my teeth*,* go to bed*,* then I get up*,* have another coffee*,* smoke a cigarette*,* get ready to go to work*,* and smoke a cigarette while getting ready.”* (Participant 08, transgender woman, 47 years old).

### Substance use

Participants reported that substance use, including alcohol and marijuana use, influenced their smoking behavior. Eleven participants reported dual use of cigarettes and marijuana. Participants also highlighted how substance use (e.g., alcohol, cocaine) often led to cigarette smoking.

*“Drugs lead you to cigarettes… most [sex workers] are cigarette and joint smokers. They mix the two*,* even up to 3 [substances including] with cocaine. I mix cigarettes and cocaine.”* (Participant 03, transgender woman, 37 years old).

Participants reported that smoking was a coping mechanism when trying to quit other substances (e.g., cocaine). Moreover, participants reported that trying to quit smoking while quitting other substances seemed overwhelming. Participants also mentioned that substance use treatment programs rarely addressed smoking cessation.

*“After quitting [crack]*,* I turned to alcohol*,* and when trying to quit alcohol*,* I turned to cigarettes. When I started the treatment [to quit alcohol]*,* they gave me medication*,* and I stopped drinking alcohol… I quit [crack]*,* but I didn’t quit cigarettes…because it was too much…”* (Participant 01, transgender woman, 43 years old).

## Social factors

Participants identified five social factors influencing cigarette smoking: (1) social influence (e.g., from peers, family, clients), (2) social acceptance of smoking, (3) social support to quit, (4) gender-based stigma, discrimination, and violence, and (5) contextual factors (e.g., work environments, hospitalization).

### Social influence

*Peers*. Three participants noted that within the TGD community, cigarette smoking is a collective behavior and a common component of social settings:

*“I have many trans friends*,* and I think there is not one person who doesn’t have a pack of cigarettes in her bag… I believe [transgender women] start smoking when they are young*,* especially at night. I think that when they are working in the forest [where they do sex work] or wherever they are*,* at night*,* standing around*,* they chat with each other*,* and time just flies by. The typical*,* ‘Oh*,* I’ll have a smoke*,* and let’s talk for a while.’ I think that life at night also leads to tobacco use.”* (Participant 15, transgender man, 33 years old).

*Family*. Eight participants highlighted the role of family members in smoking. While family members urged them to quit smoking during adolescence, this pressure diminished in adulthood. Many participants described growing up in households where smoking was common, and though it was not encouraged, it was socially accepted.

*“[Smoking] wasn’t talked about much; we just saw my dad smoking a lot.”* (Participant 10, transgender man, 47 years old).

Conversely, some participants reported that family members encouraged them not to smoke.

*“My family never gave me a positive perspective on smoking. They told me not to smoke.”* (Participant 12, transgender man 21 years old).

Three participants described the ways that sex work clients and their preferences influenced their smoking. Some participants reported limiting their smoking because clients disliked the smell. Conversely, participants reported that quitting smoking was challenging when engaging in work with clients who smoked:

*“When clients see you smoking [in the streets]*,* they don’t stop [to hire our sex services]. So*,* in the streets*,* I couldn’t smoke much.*” (Participant 08, transgender woman, 28 years old).

*“You want to quit smoking*,* you go with a client [who hired us]*,* and he lights up a cigarette in front of you… then you say ‘I can’t [quit smoking].’”* (Participant 08, transgender woman, 28 years old).

### Social acceptance of smoking

Four participants expressed that smoking is widely accepted and normalized in the TGD community and the broader society, contributing to its popularity, particularly when compared to other substances (e.g., marijuana). One participant described:

*“Culturally [in the transgender community]*,* no one tells you ‘Hey*,* stop smoking.’…It’s not like drugs. Cigarettes are [socially accepted].”* (Participant 18, transgender woman, 35 years old).

### Social support

Nine participants highlighted that social support is pivotal in quitting smoking. Moreover, they expressed a need to have stronger social networks and a supportive environment to quit smoking.

*“Human warmth is essential [to quit smoking].*” (Participant 16, transgender man, 28 years old).

*“I can’t do [quit smoking] alone. I need containment*,* support. I need to have that ear and that shoulder.”* (Participant 05, transgender woman, 47 years old).

Six participants expressed a preference for peer-led groups or interventions for smoking cessation:

*“It would be good to have a group of smokers… I would like a private group to get together on a Sunday. I think that would help people who smoke.”* (Participant 04, transgender woman, 47 years old).

### Gender-based stigma, discrimination, and violence

Stigma, discrimination, and violence related to gender identity were mentioned by seven participants as triggers for smoking. Four participants described smoking during their transition in the context of increased stigma:

*“[Gender-related violence] started at the age of thirteen*,* fourteen onwards. There was a lot of violence at home. I didn’t feel comfortable making the change [transitioning]*,* so it’s like one thing led to the other*,* [and I started smoking.]…When [people at work or school] didn’t respect how I perceived myself [or I had] family problems*,* [those] things led me to want cigarettes. I ended up addicted….”* (Participant 14, transgender man, 26 years old).

*“…There was always a lot of violence at home. To escape from that*,* I would go to the street and spent my time smoking and using drugs…. [Smoking and using drugs] were the first things I did when my uncle raped me.”* (Participant 09, transgender man, 32 years old).

### Contextual factors

*Work environments.* Seven participants reported stressful working conditions as a reason for smoking:

*“The lack of public policies*,* the limited ability to secure a [formal] job*,* and the low pay when you have to get off the street and out of prostitution—degrade us. [Society] wants to force us into low-paying jobs. These conditions are harsh and increase our anxiety*,* making smoking and everything worse. The entire context for the trans population seems designed to destroy us.“* (Participant 18, transgender woman, 35 years old).

Conversely, five participants described how formal employment contributed to smoking reduction, as a structured work environment made it harder to find time to smoke:

*“Since I started working*,* I don’t smoke as much. Work is taking me out of smoking a little bit. I can’t be asking for permission every five minutes to go out to smoke.*” (Participant 03, transgender woman, 37 years old).

*Economic factors.* Three participants reported that the economic inflation in Argentina contributed to reduced smoking, as many TGD individuals prioritized basic needs. One participant explained that economic constraints made smoking cessation pharmacotherapies unaffordable:

*“[Cigarettes] were expensive*,* so I preferred not to spend on cigarettes. I saved [money] for other things….”* (Participant 05, transgender man, 33 years old).

*“I don’t use [nicotine patches]. They are expensive*,* and I don’t have money.*” (Participant 11, transgender man, 22 years old).

*Facilitators for smoking cessation*. Six participants mentioned that although they were advised to quit smoking, particularly before gender-affirming surgeries, they were not aware of the availability or existence of trans-competent smoking cessation services:

*“I don’t know if there is a place for trans people to quit smoking.”* (Participant 05, transgender woman, 47 years old).

## Discussion

Based on a thorough literature review, we believe this is the first study to understand biopsychosocial factors influencing cigarette smoking among TGD individuals living in Argentina. Using the biobehavioral model of nicotine addiction, we identified important biological, psychological, and social factors. Biological factors included: (1) Negative impact of cigarette smoking on health (e.g., medical gender affirmation), and (2) nicotine dependence. Psychological factors included: 1) Emotions (e.g., loneliness),2) psychological disorders, 3) coping strategies (e.g., gender minority stress), 4) cognition and beliefs (e.g., psychological gender affirmation), 5) routine behavior, and 6) substance use. Social factors included: (1) social influence, (2) social acceptance of smoking, (3) social support, (4) gender-based stigma, discrimination, and violence, and (5) contextual factors (i.e., work environments).

Some of the identified factors are consistent with those of previous studies. For example, general health, medical gender affirmation, and secondhand smoke are biological factors previously identified among TGD individuals to influence cigarette smoking [5, 11, 12]. Psychological disorders [10, 21], coping strategies [12, 21, 22], routine behavior [12], and substance use [5, 9, 10, 21, 23, 24] are psychological factors previously identified among TGD individuals as well. Social influence [21], social acceptance of smoking, social support [12, 21], gender-based stigma, discrimination, and violence [6–10, 25], and work environments [6] are social factors previously identified among TGD individuals as contributors to cigarette smoking or quitting.

Our study expands current knowledge by identifying new factors that had not been previously described among TGD individuals. At the biological level, participants reported concerns about the interactions between smoking and hormone therapy. Also, participants emphasized the need for healthcare professionals to address smoking as part of gender-affirming care. At the psychological level, participants reported that smoking is part of psychological gender affirmation (e.g., smoking makes transgender men “feel more masculine”). This expands the current literature regarding the associations between smoking and gender affirmation, which has primarily focused on social, legal, and medical factors [9, 11]. At the social level, participants reported loneliness as a factor associated with smoking. Social support plays an important role in quitting smoking as interpersonal relationships often motivate behavioral changes [26]. TGD individuals often suffer from a lack of “primary group member” support, that is, the support provided by those close to them who have had similar experiences, including stigma [27, 28]. For example, in contrast to the experiences of individuals from other stigmatized groups (e.g., those who experience discrimination based on race/ethnicity, affluence), TGD individuals and their family members do not typically possess a shared stigmatized identity [27, 28]. Moreover, data suggest that a significant proportion of TGD individuals experience explicit rejection from family members because of transphobia [29–32]. Results of this study highlight how social support may increase or decrease the risk of smoking among TGD individuals and should be addressed in smoking cessation treatment.

Until 2024, Argentina had had the world’s most progressive transgender rights laws [33], such as the gender identity law and governmental job quota. Despite these laws, participants report that structural transphobia persists. Moreover, in recent years, Argentina has faced an economic crisis marked by high inflation, currency devaluation, and an increasing population living below the poverty line [34]. Social, political, and economic challenges for individuals in Argentina pose barriers to smoking cessation among TGD individuals. As noted by participants, the costs associated with nicotine replacement therapies are a barrier to smoking cessation. Moreover, trans-competent smoking cessation services are urgently needed. Previous research has identified that TGD individuals who report transphobia and stigma from healthcare providers are more likely to abandon substance abuse treatment prior to completion [35]. In contrast, TGD individuals who feel included and respected report positive treatment experiences. Smoking cessation services for TGD individuals must ensure inclusivity and respect.

### Limitations

This study has a few limitations. First, this research was limited to cigarette smoking. While the use of other nicotine and tobacco products (e.g., electronic cigarettes) is relatively low in Argentina [36], future studies are needed to understand the factors related to their use. Second, the study team was unable to recruit individuals with non-binary and other gender diverse identities. Third, given the nature of an interview, participants might have felt compelled to offer socially desirable responses. Lastly, data was self-reported and may be subject to potential recall bias.

## Conclusion

This qualitative study identified important biological, psychological, and social factors associated with cigarette smoking among TGD individuals living in Argentina. These factors have important implications in designing trans-competent smoking cessation interventions.

## Data Availability

No datasets were generated or analysed during the current study.

## Abbreviations

CAB: Community Advisory Board
LGBTQ+: lesbian, gay, bisexual, transgender, and queer
SD: standard deviation
TGD: transgender and gender diverse

## References

[1] Drope J, Schluger NW, Cahn Z, Drope J, Hamil S, Islami F et al. The tobacco atlas [Internet]. 6.^a^ ed. Atlanta Georgia: American Cancer Society and Vital Strategies; 2018. Disponible en: https://theunion.org/sites/default/files/2020-12/TobaccoAtlas_6thEdition_LoRes.pdf

[2] Instituto Nacional de Estadística y Censos (INDEC). 4° Encuesta Nacional de Factores de Riesgo. Resultados definitivos. [Internet]. INDEC. 2019 p. 217. Disponible en: https://www.indec.gob.ar/ftp/cuadros/publicaciones/enfr_2018_resultados_definitivos.pdf

[3] Linetzky B, Mejia R, Ferrante D, De Maio FG, Diez Roux AV. Socioeconomic status and tobacco consumption among adolescents: A multilevel analysis of argentina’s global youth tobacco survey. Nicotine Tob Res Septiembre De. 2012;14(9):1092–9. 10.1093/ntr/nts004 PMC3529606 22394595

[4] Pérez A, Osman A, Peña L, Abad-Vivero EN, Hardin JW, Sargent J, et al. Parent education and substance use among Latin American early adolescents. Health Behav Policy Rev 1 De Mayo De. 2018;5(3):91–9. 10.14485/HBPR.5.3.9 PMC6585987 31223629

[5] Cartujano-Barrera F, Mejia RM, Radusky PD, Cardozo N, Duarte M, Fabian S, et al. Prevalence and correlates of current cigarette smoking among transgender women in Argentina. Front Public Health Diciembre De. 2023;11:1279969. 10.3389/fpubh.2023.1279969 PMC10728477 38115852

[6] Culbreth et al. Stressors Associated with Tobacco Use Among Trans Women. 2023;8(3). Disponible en: https://www.liebertpub.com/doi/full/10.1089/trgh.2020.0168 10.1089/trgh.2020.0168 PMC10277983 37342482

[7] Lipperman-Kreda et al. Substance use among sexual and gender minorities: association with Police discrimination and Police mistrust. 2020;3(2). Disponible en: https://pubmed.ncbi.nlm.nih.gov/34651132/10.1002/sgp2.12019 PMC8513710 34651132

[8] Robertson R. Psychosocial Stressors and Tobacco Use: Views of the Transgender Community in a 2016 Focus Groups Study. En. 2018. Disponible en: https://www.ahajournals.org/doi/abs/10.1161/circ.137.suppl_1.p305

[9] Shires DA, Jaffee KD. Structural discrimination is associated with smoking status among a National sample of transgender individuals. Nicotine Tob Res Junio De. 2016;18(6):1502–8. 10.1093/ntr/ntv221 26438646

[10] Gamarel KE, Mereish EH, Manning D, Iwamoto M, Operario D, Nemoto T, Minority, Stress. Smoking patterns, and cessation attempts: findings from a Community-Sample of transgender women in the San Francisco Bay area. Nicotine Tob Res Marzo De. 2016;18(3):306–13. 10.1093/ntr/ntv066 PMC4757930 25782458

[11] Kidd et al. The relationship between tobacco use and legal document gender-marker change, hormone use, and gender-affirming surgery in a United States sample of trans-feminine and trans-masculine individuals: 2018;5(7). Disponible en: https://europepmc.org/article/MED/30334686 10.1089/lgbt.2018.0103 PMC6207148 30334686

[12] Tan AS, Gazarian PK, Darwish S, Hanby E, Farnham BC, Koroma-Coker FA et al. Smoking protective and risk factors among transgender and Gender-Expansive individuals (Project SPRING): qualitative study using digital photovoice. JMIR Public Health Surveill. 6 de octubre de 2021;7(10):e27417. 10.2196/27417 PMC8529476 34612842

[13] Berger I, Mooney-Somers J. Smoking cessation programs for lesbian, gay, bisexual, transgender, and intersex people: A Content-Based systematic review. Nicotine Tob Res 31 de agosto de 2016;ntw216. 10.1093/ntr/ntw216 27613909

[14] Hennink M, Kaiser BN. Sample sizes for saturation in qualitative research: A systematic review of empirical tests. Soc Sci Med Enero De. 2022;292:114523. 10.1016/j.socscimed.2021.114523 34785096

[15] Hiatt RA, Rimer BK. A new strategy for cancer control research. Cancer Epidemiol Biomark Prev Publ Am Assoc Cancer Res Cosponsored Am Soc Prev Oncol Noviembre De. 1999;8(11):957–64. 10566549

[16] Branstetter SA, Muscat JE, Mercincavage M. Time to first cigarette: A potential clinical screening tool for nicotine dependence. J Addict Med. 2020;14(5):409–14. 31972768 10.1097/ADM.0000000000000610 PMC7358112

[17] Heatherton TF, Kozlowski LT, Frecker RC, Fagerström KO. The Fagerström test for nicotine dependence: a revision of the Fagerström tolerance questionnaire. Br J Addict Septiembre De. 1991;86(9):1119–27. 10.1111/j.1360-0443.1991.tb01879.x 1932883

[18] Christou PA. How to use thematic analysis in qualitative research. J Qual Res Tour Diciembre De. 2022;3(2):79–95.

[19] NVivo. version 14.23.1 [Internet]. QSR International; 2023. Disponible en: https://lumivero.com/

[20] IBM SPSS Statistics for Windows (versión 29.0) [Internet]. IBM Corp. 2022. Disponible en: https://www.ibm.com/docs/en/spss-statistics/29.0.0

[21] Sun CJ, Doran KM, Sevelius JM, Bailey SR. A qualitative examination of tobacco use and smoking cessation among gender minority adults. Ann Behav Med Publ Soc Behav Med. 26 de mayo de 2023;57(7):530–40. 10.1093/abm/kaac072 PMC10312297 37232548

[22] Lee JGL, Blanchflower TM, O’Brien KF, Cofie LE, Gregory KR, Averett PE. Assessing the Potential Impact of Cigarette Packs Designed for Lesbian, Gay, Bisexual, and Transgender Adults: A Randomized Experiment to Inform, Regulation US. 2018. Health Promot Pract. enero de 2020;21(1_suppl):157S-164S. 10.1177/1524839919879923 PMC6951441 31908205

[23] Kcompt et al. Use of Cigarettes and E-Cigarettes/Vaping Among Transgender People: Results From the 2015 U.S. Transgender Survey. 2020; Disponible en: https://www.sciencedirect.com/science/article/abs/pii/S0749379720301860 10.1016/j.amepre.2020.03.027 PMC7508765 32826126

[24] Remafedi G. Lesbian, gay, bisexual, and transgender youths: who smokes, and why? Nicotine Tob Res. 2007;9(1):65–71. 17365728 10.1080/14622200601083491

[25] Wolford-Clevenger C, Hill SV, Cropsey K. Correlates of tobacco and nicotine use among transgender and gender diverse people: A systematic review guided by the minority stress model. Nicotine Tob Res 1 De Marzo De. 2022;24(4):444–52. 10.1093/ntr/ntab159 PMC11494233 34375426

[26] House JS, Landis KR, Umberson D. Social relationships and health. Sci 29 De Julio De. 1988;241(4865):540–5. 10.1126/science.3399889 3399889

[27] Thoits PA. Mechanisms linking social ties and support to physical and mental health. J Health Soc Behav Junio De. 2011;52(2):145–61. 10.1177/0022146510395592 21673143

[28] Klein A, Golub SA. Family rejection as a predictor of suicide attempts and substance misuse among transgender and gender nonconforming adults. LGBT Health Junio De. 2016;3(3):193–9. 10.1089/lgbt.2015.0111 27046450

[29] Bradford J, Reisner SL, Honnold JA, Xavier J. Experiences of transgender-Related discrimination and implications for health: results from the Virginia transgender health initiative study. Am J Public Health Octubre De. 2013;103(10):1820–9. 10.2105/AJPH.2012.300796 PMC3780721 23153142

[30] Factor RJ, Rothblum ED. A study of transgender adults and their Non-Transgender siblings on demographic characteristics, social support, and experiences of violence. J LGBT Health Res Junio De. 2007;3(3):11–30. 10.1080/15574090802092879 19042902

[31] Graham LF, Crissman HP, Tocco J, Hughes LA, Snow RC, Padilla MB. Interpersonal relationships and social support in transitioning narratives of black transgender women in Detroit. Int J Transgenderism 3 De Abril De. 2014;15(2):100–13.

[32] Koken JA, Bimbi DS, Parsons JT. Experiences of Familial acceptance–rejection among transwomen of color. J Fam Psychol. 2009;23(6):853–60. 20001144 10.1037/a0017198 PMC2840628

[33] Arístegui I, Radusky PD, Zalazar V, Romero M, Schwartz J, Sued O. Impact of the gender identity law in Argentinean transgender women. Int J Transgenderism 2 De Octubre De. 2017;18(4):446–56.

[34] Observatorio de la Deuda Social Argentina(ODSA). Deudas sociales en lista de espera. Balance social 2024 [Internet]. 2024. Disponible en: https://wadmin.uca.edu.ar/public/ckeditor/Observatorio%20Deuda%20Social/Presentaciones/2025/Observatorio_Nota_Divulgacion_Deudas_Sociales_Lista_de_Espera.pdf

[35] Lyons T, Shannon K, Pierre L, Small W, Krüsi A, Kerr T. A qualitative study of transgender individuals’ experiences in residential addiction treatment settings: stigma and inclusivity. Subst Abuse Treat Prev Policy Diciembre De. 2015;10(1):17. 10.1186/s13011-015-0015-4 PMC4432520 25948286

[36] Vital, Strategies. Tobacconomics. E-cigarettes & heated tobacco products [Internet]. The Tobacco Atlas; Disponible en: https://tobaccoatlas.org/challenges/e-cigarettes-htps/

